# Uridine Composition of the Poly-U/UC Tract of HCV RNA Defines Non-Self Recognition by RIG-I

**DOI:** 10.1371/journal.ppat.1002839

**Published:** 2012-08-02

**Authors:** Gretja Schnell, Yueh-Ming Loo, Joseph Marcotrigiano, Michael Gale

**Affiliations:** 1 Department of Immunology, University of Washington, School of Medicine, Seattle, Washington, United States of America; 2 Center for Advanced Biotechnology and Medicine, Department of Chemistry and Chemical Biology, Rutgers University, Piscataway, New Jersey, United States of America; McMaster University, Canada

## Abstract

Viral infection of mammalian cells triggers the innate immune response through non-self recognition of pathogen associated molecular patterns (PAMPs) in viral nucleic acid. Accurate PAMP discrimination is essential to avoid self recognition that can generate autoimmunity, and therefore should be facilitated by the presence of multiple motifs in a PAMP that mark it as non-self. Hepatitis C virus (HCV) RNA is recognized as non-self by RIG-I through the presence of a 5′-triphosphate (5′-ppp) on the viral RNA in association with a 3′ poly-U/UC tract. Here we define the HCV PAMP and the criteria for RIG-I non-self discrimination of HCV by examining the RNA structure-function attributes that impart PAMP function to the poly-U/UC tract. We found that the 34 nucleotide poly-uridine “core” of this sequence tract was essential for RIG-I activation, and that interspersed ribocytosine nucleotides between poly-U sequences in the RNA were required to achieve optimal RIG-I signal induction. 5′-ppp poly-U/UC RNA variants that stimulated strong RIG-I activation efficiently bound purified RIG-I protein *in vitro*, and RNA interaction with both the repressor domain and helicase domain of RIG-I was required to activate signaling. When appended to 5′-ppp RNA that lacks PAMP activity, the poly-U/UC U-core sequence conferred non-self recognition of the RNA and innate immune signaling by RIG-I. Importantly, HCV poly-U/UC RNA variants that strongly activated RIG-I signaling triggered potent anti-HCV responses *in vitro* and hepatic innate immune responses *in vivo* using a mouse model of PAMP signaling. These studies define a multi-motif PAMP signature of non-self recognition by RIG-I that incorporates a 5′-ppp with poly-uridine sequence composition and length. This HCV PAMP motif drives potent RIG-I signaling to induce the innate immune response to infection. Our studies define a basis of non-self discrimination by RIG-I and offer insights into the antiviral therapeutic potential of targeted RIG-I signaling activation.

## Author Summary

Pathogen recognition receptors (PRRs) are critical components of the innate immune response to viral pathogens, and function in the host to recognize pathogen-associated molecular patterns (PAMPs) in viral proteins or nucleic acids. Retinoic acid-inducible gene I (RIG-I) is a cytoplasmic PRR that senses viral RNA inside an infected cell. RIG-I recognizes hepatitis C virus (HCV) RNA as non-self through the presence of both a 5′-triphosphate (5′-ppp) and a 3′ poly-U/UC tract within the viral RNA. Here we examined the RNA structure-function attributes that define the HCV poly-U/UC tract as non-self to RIG-I, including nucleotide composition. We found that the 34 nucleotide poly-uridine “core” (U-core) within the HCV poly-U/UC tract RNA was required for non-self recognition by RIG-I, and interspersed ribocytosine nucleotides were also important to induce optimal RIG-I signaling. RIG-I/RNA binding studies revealed that RIG-I formed weaker interactions with HCV RNAs lacking poly-U sequences, and RNA interaction with multiple domains of RIG-I was required to activate signaling. Finally, RIG-I recognition of the U-core within the poly-U/UC tract activated anti-HCV responses *in vitro* and hepatic innate immune responses *in vivo*. Our studies identify long poly-uridine sequences with interspersed ribocytosines as an HCV PAMP motif that drives optimal RIG-I signaling.

## Introduction

Mammalian cells respond to acute virus infection through the actions of host pathogen recognition receptors (PRRs) that recognize viral pathogen-associated molecular patterns (PAMPs). The RIG-I-like receptors (RLRs) are cytoplasmic RNA helicases that function as PRRs for the recognition of RNA virus infection. The RLRs include RIG-I (retinoic acid-inducible gene I), MDA5 (melanoma differentiation-associated gene 5), and LGP2 (laboratory of genetics and physiology 2). Whereas RIG-I and MDA5 encode tandem amino-terminal caspase activation and recruitment domains (CARDs), LGP2 lacks CARDs and is thought to play a regulatory role in signaling initiated by RIG-I or MDA5 [1]. Following the recognition and binding of viral PAMP RNA, RIG-I signals through the adaptor protein mitochondrial antiviral signaling (MAVS, also known as IPS-1/VISA/Cardif) [2], [3], [4], [5]. Downstream signaling by the RLRs induces the activation of latent transcription factors, including interferon regulatory factor (IRF)-3 and NF-κB, leading to the production of type-I interferons (IFN) from the infected cell [6]. Local IFN secretion leads to the expression of hundreds of interferon-stimulated genes (ISGs) in the infected cell and surrounding tissue that mediate antiviral and immunomodulatory properties in order to restrict virus replication and impart the onset of the immune response to infection [2], [4], [6], [7], [8].

The process of RIG-I signaling activation has been revealed through structure-function studies. In addition to the N-terminal CARDs, RIG-I possesses a central DExD/H box RNA helicase/ATPase domain and a C-terminal repressor domain (RD) [6], [9]. RIG-I recognizes and binds to specific PAMP motifs within RNA marked by a free/exposed 5′-triphosphate (5′-ppp), including single-stranded (ss)RNA or double-stranded (ds)RNA [10], [11], [12], [13], [14]. RIG-I binding interactions with viral RNA are mediated through multiple contacts with the helicase domain [15], [16], [17] and the C-terminal RD, the latter of which binds 5′-ppp motifs with high specificity [13], [18], [19]. RIG-I recognition and binding of viral RNA relieves auto-repression and drives ATP hydrolysis and conformational rearrangements that expose the CARDs for downstream signaling to initiate the immune response to infection [9], [13], [18]. Despite the recent advancements in RIG-I structural biology [15], [16], [17], [20], the nature of RIG-I recognition of sequence-specific PAMP RNA motifs remains unclear. Accurate discrimination of self from non-self by PRRs is essential to avoid immune triggering against self that leads to autoimmunity [21], [22], [23]. In this sense, PRR recognition of a single PAMP motif alone, such as 5′-ppp, within viral RNA is unlikely to accurately discriminate the comparably low abundance PAMP RNA from the high abundance host RNA. Moreover, the presence of a single motif within host RNA that displays the PAMP signature could induce aberrant signaling against self, whereas a combinatorial non-self signature for PRR binding and signaling activation would serve to accurately discriminate it as a PAMP. Previous studies have revealed that multiple parameters define an RNA PAMP for RIG-I recognition, including 5′-ppp [10], [11], [14], length (>19 nt) [17], [24], secondary structure characteristics [12], [25], and nucleotide sequence motifs [26], [27].

RIG-I is essential for host cell recognition of a variety of RNA viruses [28], [29], including hepatitis C virus (HCV). HCV is a positive-sense ssRNA virus that replicates in hepatocytes and causes chronic liver disease and liver cancer. Approximately 200 million people worldwide are persistently infected with HCV [30], [31], [32], and infection is characterized by chronic viral replication, producing viral RNA that can trigger innate immune responses [9], [33]. HCV RNA is recognized as non-self by RIG-I [33] through recognition of the poly-U/UC tract located in the 3′ non-translated region (NTR) of the viral genomic RNA, thus defining the poly-U/UC tract as a PAMP motif of HCV [26]. The HCV poly-U/UC tract is approximately 100 nucleotides (nt) in length and is essential for virus replication and viability [34], [35], [36]. While the poly-U/UC tract is conserved in 3′ NTR placement within all genotypes and strains of HCV, it varies in the length of poly-uridine sequences and the positioning of ribocytosine nucleotides. RIG-I recognition of the HCV poly-U/UC tract is dependent on the 5′-ppp RNA length and sequence composition [26], [27], and RIG-I signaling is attenuated in response to HCV poly-U/UC RNAs shorter than 50 nucleotides in length or with a reduced poly-uridine nucleotide composition compared to wild-type viral RNA [26]. Although RIG-I recognizes HCV RNA, the specific RNA sequence motifs in the HCV poly-U/UC tract that confer RIG-I recognition are not known.

In this study, we evaluate the properties of RIG-I recognition of HCV RNA by conducting a detailed structure-function analysis of RIG-I and poly-U/UC RNA interactions and innate immune signaling. Our results show that the poly-uridine core (U-core) within the HCV poly-U/UC tract was essential for recognition by RIG-I, indicating that RIG-I recognizes long poly-uridine regions as non-self motifs within 5′-ppp RNA. In addition, we found that the affinity of RIG-I/RNA binding interactions, and critical contacts between the PAMP RNA and RIG-I helicase domain, both defined an HCV RNA recognition sequence in which long poly-uridine sequences (>U17) with interspersed ribocytosines induced *in vitro* anti-HCV responses and hepatic innate immune responses *in vivo*. Thus, RIG-I recognition of the U-core within the poly-U/UC tract of the 5′-ppp HCV RNA is the trigger of innate antiviral immunity to HCV infection. Poly-uridine sequences could thereby offer a novel application for innate immune stimulation in vaccine vectors and antiviral therapeutic strategies for controlling RNA virus infection.

## Results

### The U-core of the HCV poly-U/UC tract is required for RIG-I signal induction

**Figure 1:**
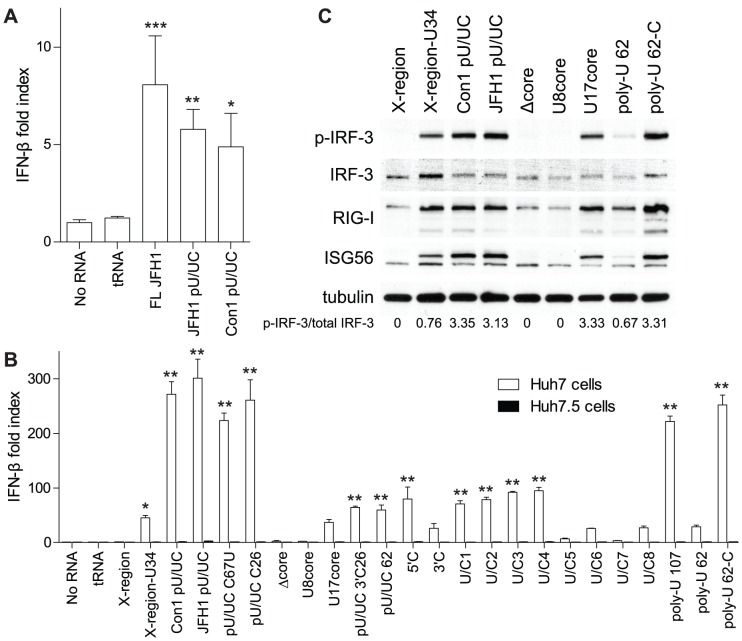
HCV poly-U/UC RNA constructs activate RIG-I signaling. A) Induction of the IFN-β-promoter in Huh7 cells transfected with equal moles of tRNA, full-length JFH1, JFH1 pU/UC, or Con1 pU/UC RNA. IFN-β-promoter luciferase activity is shown as mean IFN-β fold index (compared to cells with No RNA, ± s.d. for three replicates). Huh7 cells were transfected with the various RNA constructs and 16 hours later cells were harvested for dual luciferase activity. Asterisks indicate a significant difference compared to No RNA control as determined by a one-way ANOVA adjusted with Bonferroni's multiple comparison test (*P<0.05, **P<0.01, ***P<0.001). B) Induction of the IFN-β-promoter in Huh7 or Huh7.5 cells transfected with 350 ng of the indicated RNA constructs. IFN-β-promoter luciferase activity is shown as the mean IFN-β fold index ± s.d. for three replicates, and data was normalized to the No RNA control. Cells were harvested for dual luciferase activity 16 hours post-RNA transfection. Asterisks indicate a significant difference compared to No RNA control as determined by a one-way ANOVA adjusted with Bonferroni's multiple comparison test (*P<0.05, **P<0.001). C) The abundance of phospho-IRF-3 (Ser396), total IRF-3, RIG-I, ISG56, and tubulin were measured by immunoblot. Huh7 cells were transfected with the indicated RNA constructs and cells were harvested for protein analysis 16 hours later. RIG-I and ISG56 are IFN-β-stimulated genes. The ratio of phospho-IRF-3/total IRF-3 was calculated by measuring the relative immunoblot band intensities using ImageJ software (NIH). Data shown in all panels are representative of three independent experiments.

To determine the RNA sequence elements in the HCV poly-U/UC tract required for non-self recognition by RIG-I, we developed multiple poly-U/UC RNA constructs encoding changes in distinct regions termed the 5′arm, U-core, and 3′arm, and based on the HCV genotype 1b consensus (Con1) poly-U/UC sequence (Table 1). We also developed two RNA constructs encoding either the HCV Con1 3′ NTR X-region sequence alone, or including a 34 nt poly-uridine sequence between stem-loops 1 (SLI) and 2 (SLII) of the X-region [36], [37] (X-region-U34 RNA construct; see Table 1 legend). Each RNA was generated *in vitro* from a DNA template using the T7 RNA polymerase, which resulted in the RNA products having a 5′-ppp and three guanine nt at the 5′ end [38]. Previous studies demonstrated that the poly-U/UC tract, but not the X-region, of the HCV 3′ NTR was responsible for RIG-I recognition and triggering of innate immune signaling [26]. We assessed the ability of equal moles of 5′-ppp full-length JFH1 HCV RNA or the JFH1 poly-U/UC tract to activate RIG-I signaling to the IFN-β-promoter in human hepatoma (Huh7) cells harboring an intact RIG-I pathway. The full-length HCV RNA genome and the poly-U/UC tract RNA from either genotype 1b (Con1) or 2a (JFH1) were able to activate signaling and drive transcription from the IFN-β-promoter (Figure 1A), confirming that the poly-U/UC tract of the HCV RNA is a PAMP that triggers PRR signaling. We also found that induction of the IFN-β-promoter by the Con1 and JFH1 poly-U/UC tract (pU/UC) RNAs in Huh7 cells (Figure 1B) was linked with the induction of IRF-3 phosphorylation and ISG expression (Figure 1C). Additionally, Huh7.5 cells that lack a functional RIG-I pathway [33], [39] failed to respond to transfected HCV RNA constructs (Figure 1B), thus defining RIG-I-dependence to HCV PAMP recognition and innate immune signaling.

**Table 1:** HCV poly-U/UC RNA constructs developed for RIG-I binding and activation studies.

RNA construct^a^	5′ arm^b^	U-core	3′ arm
Con1 pU/UC	5′GGCCAUCCUGUUUUUUUCCC(U11)C	U34	CUCCUUUUUUUUUCCUCUUUUUUUCCUUUUCUUUCCUUU
JFH1 pU/UC	5′ACUGUUCC	U43	C(U14)CCCUCUUUCUUCCCUUCUCAUCUUAUUCUACUUUCUUUCUU
pU/UC C26	5′GGCCAUCCUGUUUUUUUCCC(U11)C	U34	CUCCUUUUUUUUUCCUCUUUUUUUCCUUUUCUUUCCUUU(C26)
pU/UC 3′C26	5′GGCCAUCCUGUUUUUUUCCC(U11)C	U34	CUCCUUUUUUUUUCCCCCCCCCCCCCCCCCCCCCCCCCC
pU/UC C67U	5′GGCCAUCCUGUUUUUUUCCC(U11)C	U34	UUCCUUUUUUUUUCCUCUUUUUUUCCUUUUCUUUCCUUU
poly-U 107	5′UUUUUUUUUUUUUUUUUUUU(U11)U	U34	UUUUUUUUUUUUUUUUUUUUUUUUUUUUUUUUUUUUUUU
Δcore	5′GGCCAUCCUGUUUUUUUCCC(U11)C	---	CUCCUUUUUUUUUCCUCUUUUUUUCCUUUUCUUUCCUUU
U8core	5′GGCCAUCCUGUUUUUUUCCC(U11)C	U8	CUCCUUUUUUUUUCCUCUUUUUUUCCUUUUCUUUCCUUU(C26)
U17core	5′GGCCAUCCUGUUUUUUUCCC(U11)C	U17	CUCCUUUUUUUUUCCUCUUUUUUUCCUUUUCUUUCCUUU(C17)
pU/UC 62	5′GGCCAUCCUG----------------	U34	CUCCUUUUUUUUUCCUCU---------------------
5′C	5′CCCCCCCCCC----------------	U34	CUCCUUUUUUUUUCCUCU---------------------
3′C	5′GGCCAUCCUG----------------	U34	CCCCCCCCCCCCCCCCCC---------------------
poly-U 62	5′UUUUUUUUUU----------------	U34	UUUUUUUUUUUUUUUUUU---------------------
poly-U 62-C	5′UUUUUUUUUU----------------	U34	CUUUUUUUUUUUUUUUUU---------------------
U/C1	5′UUCCUUCCUU----------------	U34	CUCCUUUUUUUUUCCUCU---------------------
U/C2	5′UUUUUUUUUG----------------	U34	CUCCUUUUUUUUUCCUCU---------------------
U/C3	5′UUUUUUUUUG----------------	U34	CUUUUUUUUUUUUCCUUU---------------------
U/C4	5′GGUUUUCCUU----------------	U34	CUUUUUUUUUUUUUUUUU---------------------
U/C5	5′GGCCAUCCUG----------------	U10	C(U10)C(U10)CUCUCCUUUUUUUUUCCUCU-------
U/C6	5′GGCCAUCCUG----------------	U15	C(U15)CUUCUCCUUUUUUUUUCCUCU------------
U/C7	5′GGCCAUCCUG----------------	U10	CCC(U10)CCC(U8)CUCCUUUUUUUUUCCUCU------
U/C8	5′GGCCAUCCUG----------------	U18	CCCCCC(U10)CUCCUUUUUUUUUCCUCU----------

a The X-region RNA construct has the sequence 5′-GGUGGCUCCAUCUUAGCCCUAGUCACGGCUAGCUGUGAAAGGUCCGUGAGCCGCUUGACUGCAGAGAGUGCUGAUACUGGCCUCUCUGCAGAUCAAGU-3′. The X-region-U34 RNA construct has the sequence 5′-GGGUGGCUCCAUCUUAGCCCUAGUCACGGCUAGCUGUGAAAGGUCCGUGAGC(U34)CGCUUGACUGCAGAGAGUGCUGAUACUGGCCUCUCUGCAGAUCAAGU-3′.

b All RNAs include a 5′-ppp and three guanine nucleotides at the 5′ end of the RNA. Dashes indicate nucleotide deletions, and underlined nucleotides show changes from the HCV Con1 poly-U/UC sequence. Long homo-polymeric nucleotide sequences are indicated in parentheses with the nucleotide designation followed by the number of nucleotides in the sequence.

We further evaluated Huh7 and Huh7.5 cell responses to HCV poly-U/UC tract RNA construct derivatives (Table 1 and Figure 1). Neither the C67U nt substitution (pU/UC C67U construct) nor the addition of 26 ribocytosine nucleotides to the 3′end of the RNA (pU/UC C26) significantly changed signaling compared to wild-type poly-U/UC RNA in Huh7 cells. However, despite the presence of a 5′-ppp, deletion of the U-core from the pU/UC tract (Δcore) ablated the induction of IRF-3 phosphorylation and signaling to the IFN-β-promoter, thus preventing ISG expression (Figure 1B and 1C). Replacement of the U-core with 8 uridine nts (U8core) failed to restore PAMP recognition by RIG-I, whereas a 17 uridine nt core RNA (U17core) triggered weak signaling activity, indicating that in addition to a 5′-ppp, a minimum U-core length of approximately 17 uridine nucleotides is required for RIG-I recognition of the HCV poly-U/UC tract. In addition, while the 5′-ppp HCV X-region RNA did not trigger RIG-I signaling, we found that insertion of a 34 nt poly-U sequence (U34) into the X-region RNA between SLI and SLII (X-region-U34) resulted in its recognition as a PAMP to stimulate IRF-3 phosphorylation, IFN-β-promoter activity, and ISG expression (Figure 1B and 1C). Other RNA constructs with sequence changes in the U-core region (U/C5, U/C7) exhibited a significantly decreased capacity to stimulate induction of the IFN-β-promoter (P<0.01 and P<0.001 respectively, two-tailed Student's t-test) compared to either the wild-type Con1 pU/UC RNA or the truncated pU/UC 62 RNA (encoding the wild-type U-core). Taken together, these results demonstrate that the U-core of the HCV poly-U/UC tract is required for non-self recognition by RIG-I and subsequent activation of the innate immune response.

Analysis of specific poly-U/UC tract constructs also revealed that interspersed ribocytosine nucleotides between the poly-U sequences in the RNA were necessary to achieve optimal RIG-I signal induction. We noticed that substitution of the last 26 nucleotides in the 3′arm of the poly-U/UC tract to ribocytosines (pU/UC 3′C26) resulted in a significant decrease in IFN-β-promoter induction compared to the wild-type Con1 pU/UC sequence (Figure 1B; P = 0.0047, two-tailed Student's t-test). Thus, although the U-core is required for poly-U/UC recognition by RIG-I, the uridine/cytosine sequences located in the 3′arm play a role in PAMP recognition. We therefore developed a set of truncated poly-U/UC constructs with C to U substitutions and compared their PAMP activity, defined as signaling of IFN-β-promoter induction, with PAMP activity driven by the equivalent-length pU/UC 62 construct. The 5′C, 3′C, U/C1, U/C2, U/C3, and U/C4 RNA constructs each exhibited similar PAMP activity as the pU/UC 62 RNA to induce the IFN-β-promoter (Figure 1B). Removal of all ribocytosine nucleotides revealed a property of length-dependent, U-specific PAMP activity of which the shorter poly-U 62 RNA induced significantly less signaling to the IFN-β-promoter compared to the longer poly-U 107 RNA in Huh7 cells (Figure 1B; P = 0.0004, two-tailed Student's t-test). However, this effect of RNA length was overcome by inserting a single ribocytosine nucleotide into the poly-U 62 RNA, wherein the poly-U 62-C RNA was able to drive IRF-3 phosphorylation, IFN-β-promoter induction, and ISG expression as efficiently as the wild-type Con1 pU/UC RNA (Figure 1B and 1C). Thus, poly-U length and ribocytosine content impact RIG-I recognition and PAMP activity of the HCV poly-U/UC tract.

### RNA binding interactions determine RIG-I signaling activation

**Figure 2:**
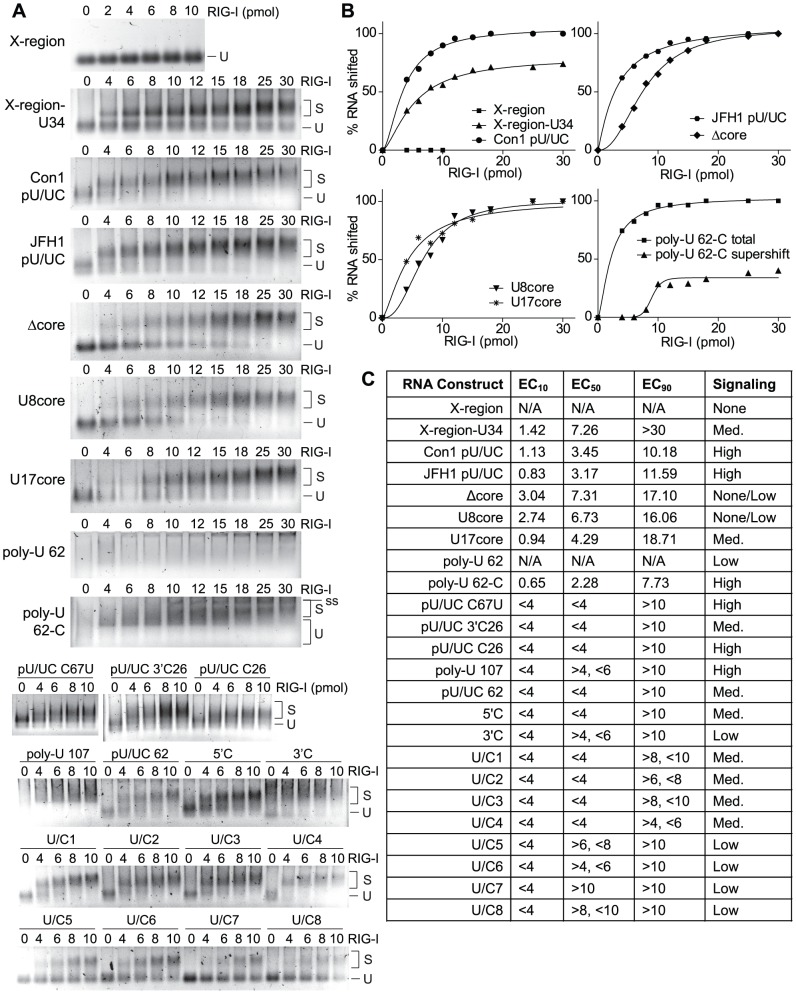
Differential binding between poly-U/UC RNA constructs and purified RIG-I protein *in vitro*. A) EMSA gel-shift assays. RNA (10 pmol) was incubated with increasing concentrations of purified recombinant RIG-I protein (0–30 pmol), then complexes were separated on native agarose gels and RNA was visualized using SYBR Gold nucleic acid stain. Unshifted RNA, U; shifted RNA/protein complex, S; supershifted RNA/protein complex, ss. B) RIG-I/RNA binding curves were generated from the gel-shift analyses and plotted for the X-region, X-region-U34, Con1 pU/UC, JFH1 pU/UC, Δcore, U8core, U17core, and poly-U 62-C RNAs. Due to poor band formation of the poly-U 62 RNA on the non-denaturing gel used in our EMSA, we were unable to conduct a gel-shift analysis of this particular RNA. C) Table comparing the effective pmol concentration of RIG-I required to shift 10% (EC_10_), 50% (EC_50_), or 90% (EC_90_) of each RNA construct. Not applicable, N/A. RIG-I signaling for each RNA construct was determined in Figure 1B, and the magnitude of the signaling activity is listed in the table as either None/Low (IFN-β fold index = 0–3), Low (IFN-β fold index = 3–30), Med. (IFN-β fold index = 30–100), or High (IFN-β fold index >100).

To examine the binding interactions between HCV poly-U/UC RNA and RIG-I that impart PAMP activity, we first conducted an electrophoretic mobility gel-shift assay (EMSA) with purified recombinant RIG-I protein and 10 pmol of each poly-U/UC RNA construct (Figure 2A). Comprehensive EMSA analyses were conducted on nine RNA constructs to generate saturation-binding curves (Figure 2B). The largest differences in RIG-I binding between the various poly-U/UC RNA constructs were detected at lower RIG-I concentrations (0–10 pmol), whereas we observed increasing saturation of RIG-I/RNA binding reactions for most RNA constructs at concentrations of RIG-I that exceeded 10 pmol. In order to compare the association differences between the RNA constructs and RIG-I, we calculated the pmol effective concentration (EC) of RIG-I required to shift 10% (EC_10_), 50% (EC_50_), or 90% (EC_90_) of each RNA as determined by EMSA (Figure 2C). The EC values for the X-region, X-region-U34, Con1 pU/UC, JFH1 pU/UC, Δcore, U8core, U17core, and poly-U 62-C RNAs were calculated using the graphical equation generated from the comprehensive EMSA analyses (graphically depicted in Figure 2B). The remaining RNA constructs had more limited EMSA analyses; therefore, we were only able to calculate the general ranges for the EC values to between 0–10 pmol RIG-I.

As shown in Figure 2C, the EC_10_ values for the various poly-U/UC RNA constructs ranged from 0.65–3.04 pmol RIG-I, and EC_50_ values ranged from 2.28–7.31 pmol RIG-I. Noting that all RNAs included a 5′-ppp, we found that the X-region RNA did not bind to RIG-I in the range of 0–10 pmol of protein, while the X-region-U34 RNA had an EC_10_ value of 1.42 pmol RIG-I and >50% of the RNA bound to 8 pmol of RIG-I, demonstrating that the inserted U34 sequence promoted stable binding interactions between RIG-I and the X-region-U34 RNA. In addition, we found that EC_10_ values were significantly larger (P = 0.0005, two-tailed Student's t-test) for RNAs that did not signal (Δcore, U8core) versus RNAs that demonstrated PAMP activity and signaled to the IFN-β-promoter (X-region-U34, Con1 pU/UC, JFH1 pU/UC, U17core, and poly-U 62-C). The non-signaling Δcore and U8core RNAs both contain large deletions in the U-core region, while the RNAs that induced RIG-I signaling all contained poly-U sequences >17 nt in length. These data reveal that RIG-I forms only weak interactions with 5′-ppp RNAs lacking poly-U sequences, and demonstrate that the 34 nt poly-U core of the HCV pU/UC tract is required to confer stable RIG-I/RNA binding interactions. There was not a significant difference in the EC_50_ or EC_90_ values between the signaling and non-signaling RNAs, indicating that differences in RNA affinity may play an important role in determining RIG-I signaling activation at lower, more physiologically relevant concentrations of RIG-I.

We also examined RIG-I/RNA affinity among RNAs denoted as non-signaling/low signaling (Δcore, U8core), medium signaling (X-region-U34, U17core), and high signaling (Con1 pU/UC, JFH1 pU/UC, poly-U 62-C) based on their ability to induce different levels of signaling to the IFN-β promoter. There was a statistically significant increase in the EC_10_ (P = 0.0025, two-tailed Student's t-test) and EC_50_ (P = 0.004) values measured for no/low signaling RNAs compared to high signaling RNAs, thus demonstrating that the Δcore and U8core RNAs (lacking a poly-U core) form significantly weaker interactions with RIG-I than those RNAs with stronger PAMP activity. A significant difference was also detected when comparing the EC_10_ values measured for no/low signaling compared to medium signaling RNAs (P = 0.0263). In general, the EC_90_ for all RNA constructs was >10 pmol RIG-I regardless of whether or not the RNA could drive RIG-I activation. However, there was still a significant difference in the EC_90_ values between RNAs that did not signal (Δcore, U8core) and the RNAs that induced high signaling to the IFN-β-promoter (Con1 pU/UC, JFH1 pU/UC, poly-U 62-C; P = 0.0208 using a two-tailed Student's t-test). Thus, our data link HCV RNA sequences containing poly-U motifs with stronger RIG-I binding and enhanced signaling for an overall potent PAMP activity. Taken together, our data indicate that the strength of RIG-I/RNA binding interactions defines an HCV RNA recognition sequence in which long poly-uridine sequences (>U17) with interspersed ribocytosines drive optimal RIG-I binding and signaling to induce the innate immune response.

### RNA interactions with the RIG-I RD and helicase domain are important for PAMP signaling

**Figure 3:**
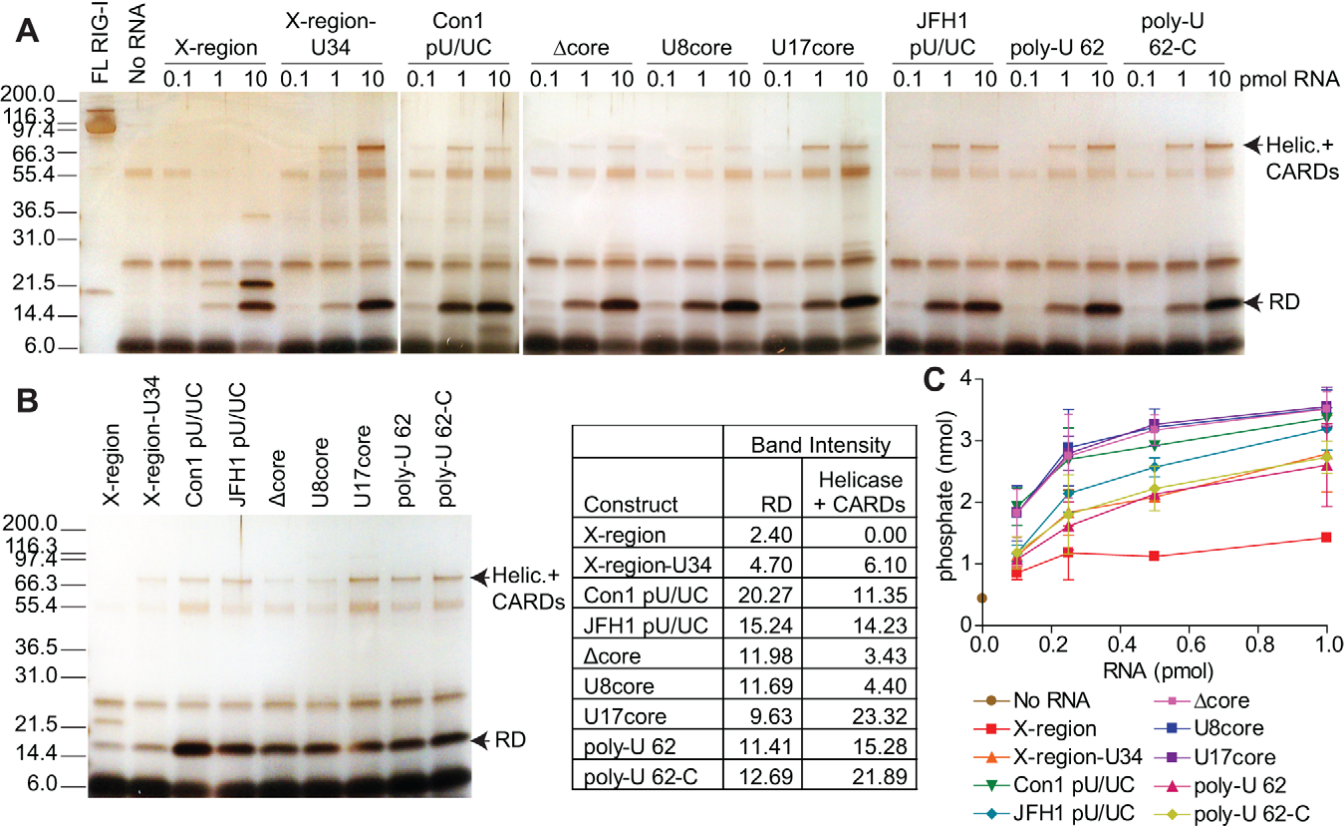
HCV poly-U/UC RNA constructs interact with the RIG-I RD and helicase domain. A) Limited-trypsin proteolysis of 30 pmol purified RIG-I with increasing amounts of RNA. Repressor domain, RD; helicase domain and CARDs, Helic. +CARDs. B) Limited trypsin proteolysis of 30 pmol purified RIG-I protein with 1.0 pmol of each indicated RNA construct. RIG-I digestion products were separated on the same gel and relative band intensities (listed as % of total) were measured using ImageJ gel imaging software (NIH). C) ATPase activity of purified RIG-I protein incubated with increasing amounts of RNA. Data shown are means ± s.d. for two replicates.

RIG-I is maintained in an auto-repressed conformation in uninfected cells where the CARDs interact with either the C-terminal repressor domain (RD), the helicase insertion domain (Hel2i), or both domains [9], [16], [17]. Crystal structure studies of RIG-I bound to RNA have revealed that the RD interacts with the 5′-ppp terminus of the RNA [14], [18], [19], which brings RNA structures in close contact with the helicase domains to allow for more specific RIG-I/RNA interactions [15]. To further assess RNA interactions with RIG-I, and to determine how HCV PAMP RNA interacts with the helicase domain and RD of RIG-I, we conducted limited-trypsin proteolysis of RIG-I/RNA complexes for nine RNA constructs selected to represent a range of PAMP activity (Figure 3). RIG-I is highly sensitive to trypsin proteolysis in the absence of PAMP RNA; however, upon binding to an RNA ligand RIG-I undergoes conformational changes that protect the RD and other RNA-bound domains from trypsin digestion [9], [26].

We found that all RNA constructs were able to bind and protect the RIG-I RD, albeit variably, from trypsin proteolysis in a dose-dependent manner (Figure 3A). While we previously saw no binding between RIG-I and the X-region RNA in our EMSA analysis, the trypsin proteolysis assay revealed limited protection of the RIG-I RD by the X-region RNA. These differences in X-region binding likely reflect the higher amount of RIG-I required for protein visualization in the limited-trypsin proteolysis assay, and indicate that some degree of background binding occurs between RIG-I and ligand RNA in this assay. There was not a significant difference in the RD band intensity when comparing RIG-I protection from 1 pmol of non-signaling (X-region, Δcore, U8core) versus signaling RNAs, likely reflecting binding supported by the 5′-ppp on all of the RNAs (Figure 3B) [10], [11]. However, we detected a dose-dependent protection of an approximately 78 kDa proteolytic product representing a large portion of the RIG-I helicase domain and CARDs (Figure 3A). This product was only detected for RNA constructs that exhibited PAMP activity to induce signaling to the IFN-β-promoter (X-region-U34, Con1 pU/UC, JFH1 pU/UC, U17core, poly-U 62, poly-U 62-C). In addition, there was a statistically significant difference in the helicase+CARDs band intensity when comparing protease protection of RIG-I resulting from 1 pmol of non-signaling versus signaling RNA (Figure 3B; P = 0.0147, two-tailed Student's t-test). These observations suggest that in addition to RD interactions, RNA interactions with the helicase domain may be required for induction of RIG-I signaling. We did not detect a significant difference in RIG-I ATPase activity when bound to the various RNA constructs, but the lowest ATPase activity was observed for RIG-I bound to the X-region RNA (Figure 3C). Taken together, these results indicate that RNA binding interactions with the RIG-I RD are mediated by the 5′-ppp [10], [14], [18], [19], and specific interactions between the helicase domain and poly-uridine tract of the HCV RNA are required for PAMP activity and to initiate RIG-I signaling.

### HCV poly-U/UC RNA variants trigger differential anti-HCV and hepatic innate immune responses

**Figure 4:**
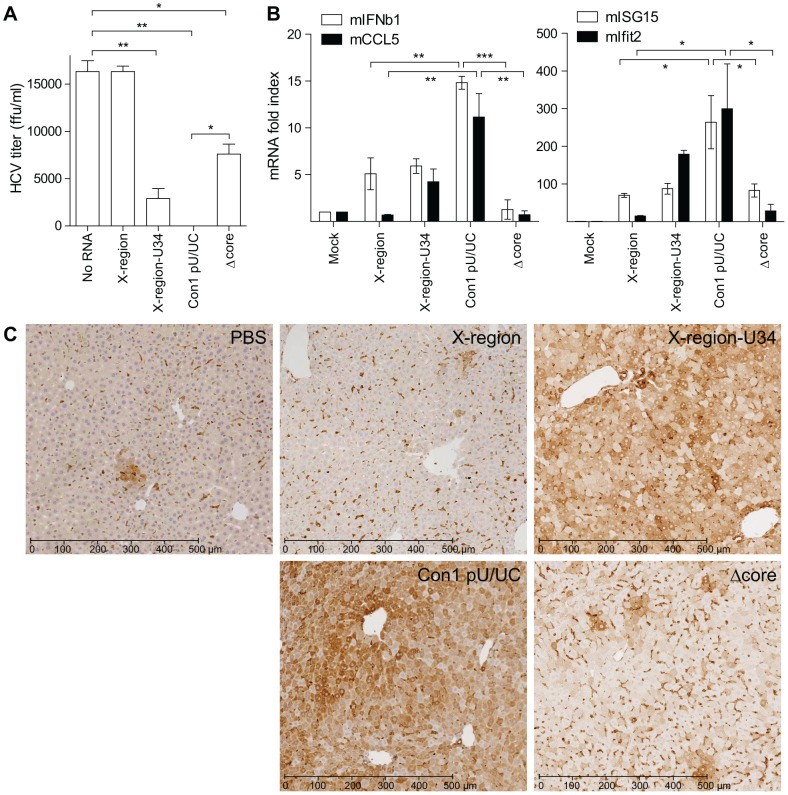
HCV poly-U/UC RNA variants trigger differential anti-HCV and hepatic innate immune responses. A) Huh7 cells were transfected with the indicated poly-U/UC RNA constructs 12 hours prior to HCV infection (MOI = 0.1), and virus production was assessed 48 hours post-infection. Data shown are means ± s.d. for three replicates. Asterisks indicate a significant difference compared to No RNA control as determined by a one-way ANOVA adjusted with Bonferroni's multiple comparison test (*P<0.001, **P≤0.0001). B) Wild-type mice (n = 2) received 200 µg of X-region RNA, X-region-U34 RNA, Con1 pU/UC RNA, or Δcore RNA. Mock-transfected wild-type mice (n = 1) received PBS. Comparative measurements of hepatic mRNA and protein expression were measured 8 hours post-transfection. Real-time quantitative PCR was performed to examine expression of *IFN-β*, *CCL5*, *Ifit2*, *ISG15*, and *GAPDH*. Results were normalized to the expression of mouse *GAPDH* mRNA, and mRNA fold index was normalized to Mock controls. Data shown are means ± s.d. for two replicates, and gene expression data was confirmed by two independent real-time PCR analyses. Asterisks indicate a significant difference as determined by a one-way ANOVA adjusted with Bonferroni's multiple comparison test (*P<0.05, **P<0.01, ***P<0.001). C) Following RNA transfection, mouse livers were recovered and immunohistochemistry staining was conducted for mouse ISG54. The black scale bar indicates a distance of 500 µm.

To determine how the poly-U core sequence imposes PAMP activity that initiates RIG-I signaling of innate immunity, we measured HCV production in Huh7 cells that were first transfected with poly-U/UC RNA constructs to stimulate RIG-I signaling (Figure 4A; No RNA, X-region, X-region-U34, Con1 pU/UC, and Δcore). 12 hours following RNA transfection, the cells were infected with HCV and virus production was then assessed 48 hours later. We found that X-region RNA did not stimulate cellular suppression of HCV infection, whereas the Con1 pU/UC RNA stimulated a potent innate immune response that significantly suppressed HCV infection as compared to non-transfected control cultures (Figure 4A). These analyses were applied to a non-parametric correlation test to reveal an inverse correlation between HCV production titer (ffu/ml) in the transfected cells and PAMP activity of the different RNAs as measured by the IFN-β-promoter fold index in Huh7 cells (Spearman r = −0.97; two-tailed P-value = 0.0167). Overall, RNA constructs that induced higher levels of IRF-3 phosphorylation and IFN-β-promoter activity (X-region-U34, Con1 pU/UC; Figure 1) were more effective in inducing RIG-I signaling to suppress HCV infection.

To determine if the differential PAMP activity of HCV RNA constructs imposed different levels of hepatic innate immune signaling and response *in vivo*, we examined the ability of the constructs to trigger hepatic innate immune responses using an intraperitoneal injection model in mice. This model recapitulates RIG-I-dependent PAMP signaling of hepatic innate immunity triggered by HCV RNA [26]. Four poly-U/UC RNA variants (X-region, X-region-U34, Con1 pU/UC, and Δcore) were mixed with a lipid-based *in vivo* RNA transfection reagent and injected into wild-type C57BL/6 mice, and livers were collected 8 hrs later for assessment of the hepatic innate immune response. Expression of *IFN-β* mRNA and several interferon-stimulated genes (ISGs) were measured using real-time quantitative PCR (Figure 4B). We found that hepatic *IFN-β*, *CCL5*, *ISG15*, and *Ifit2* (also known as ISG54) mRNA expression was significantly higher in mice transfected with Con1 pU/UC RNA compared to either X-region or Δcore RNA, indicating that the U34-core of the pU/UC tract is required for PAMP activity that induces the expression of IFN-β and other ISGs. Expression of hepatic *Ifit2* mRNA was also higher in mice transfected with X-region-U34 RNA compared to X-region RNA, although the difference in gene expression did not reach statistical significance, implicating the requirement of poly-U RNA sequences for activation of hepatic innate immune responses *in vivo*. Expression of *Ifit2* also correlated with ISG54 protein expression as shown by immunohistochemical staining of mouse liver sections (Figure 4C). ISG54 protein expression was substantially higher in mice that received X-region-U34 or Con1 pU/UC RNA compared to either PBS, X-region, or Δcore RNA. Taken together, these studies indicate that the 34 nt poly-U core of the HCV poly-U/UC tract is required for PAMP activity *in vivo* to drive RIG-I signaling of the hepatic innate immune response.

### HCV poly-U/UC sequence variability

We examined genetic variability of the poly-U/UC tract in HCV genome sequences containing full coverage of the 3′NTR that were available from the GenBank sequence database. Non-redundant HCV poly-U/UC sequences were aligned to examine sequence variability. We found that the poly-U/UC sequences varied in both length and nucleotide (nt) composition (see Table 2). In terms of length, the 5′arm ranged from 8–42 nt, the U-core ranged from 12–96 nts, and the 3′arm ranged from 0–80 nt. Nine pU/UC sequences contained a U-core with fewer than 20 uridine nucleotides, suggesting that these genomes likely have low RIG-I signaling activity. In general, HCV pU/UC genotype 1 sequences contained fewer purine nucleotides than genotype 2 sequences. Within the 5′arm of the pU/UC tract, genotype 1 nucleotide composition ranged from 12–44% purines compared to 16.7–50% purines in genotype 2 sequences. Within the 3′arm of the pU/UC tract, 41% of the genotype 1 sequences lacked purine nts (range of 0–11.6% purine composition), whereas genotype 2 sequence composition ranged between 5–11% purine nts in the 3′arm. We were unable to examine pU/UC sequence variability for genotypes 3–6 due to a deficiency of full-length 3′NTR sequences from those viral genotypes in the available sequence databases. Our analysis of HCV genome sequences reveal nt composition and length variability in the poly-U/UC tract of the HCV genome, and importantly within the U-core, suggesting that PAMP activity may differ substantially between HCV genotypes and within patient quasispecies populations.

**Table 2:** HCV poly-U/UC sequence variability.

GenBank Acc. Gen.^a^	5′ arm^b^	U-core	3′ arm^c^
AJ238799.1b	5′GGCCAUCCUGUUUUUUUCCC(U11)C	U34	CUCCUUUUUUUUUCCUCUUUUUUUCCUUUUCUUUCCUUU
AB001040.1b	5′GGCCAUUC	U16	CUUUCUUCUUU
AB016785.1b	5′GGCCGUCCUG	U18	
AB049088.1b	5′GGCCAUUCCC	U81	CUCUUCUUUUCUUUAUUCCUUCUUU
AB049089.1b	5′GGCCGUUCC	U64	CUUUUCCCCUUUUUUAUUUUUCUUUCUU
AB049090.1b	5′GGCCAUCCUGUUUUUUUGUUUUUUC	U43	CUUUUUUUCCCUUUUUUUUAUUUUAUUUUCUUUUGGU
AB049091.1b	5′GGCCAUCCCCC	U96	CCUCUUUUUUUCCUUUUCUUCUUU
AB049095.1b	5′GGCCGUUCUG	U85	CCUUUUUUUUAUUCCUCUUCU
AB049101.1b	5′GGCCAUCCCCUUUG	U94	AUUUCUCCUUCUUUU
AB080299.1b	5′GGCCAUUCCU	U24	CUUUUUUUUUCC(U24)CCUUUUCUUUCUUCUUU
AB249644.1b	5′GGCCAUUUUC	U14	CUUCUUUCUUUUUCUUUUUCUUUUUUUCCUUCUUU
AF054247.1b	5′AGCCAUUUCCUG	U28	CUUUUUUUUUUUCUUUCCUUUCCUUCUUUUUUUCCUUUCUUUUUCCCUUCUUUAAU
AF139594.1b	5′GGCCAUUUCCUG(U15)GG	U39	CCUUUCCUUCUUUUUUUUUUUUUCCCUCUUUAU
AF333324.1b	5′GGCCAUUUCCUG	U34	CUUUUCCUUCUUUUUCCCUUUUUCUUUCUUCCUUCUUUAAU
AF176573.1b	5′GGCCAUCCUGUG	U75	AUUUCCUUUUCUU
AF356827.1b	5′GGCCAACCUG(U26)CC	U34	CCUUUUUUUCUUUUUUUUUUUUUUUUUCCUUCCUUUU
AJ132997.1b	5′GGCCAUCCUG	U16	CUUUCUUU
AY460204.1b	5′GGCCAUUUUUCC	U23	CUUUUUUUUUUCCUUUUUUUCUUUUUUUUUCUUUUCUUU
D85018.1b	5′GGCCAUUC	U35	CGUUUCUUUUUCUUCUUUUUGUUUUCUCUUCUCCUUUU
D85021.1b	5′GGCCAUUCCCC(U14)CCGC	U33	CUUUUUUUUUCC(U27)CUUUUU
D85022.1b	5′GGCCAUCCCCC(U13)CCGC	U21	CUUUUUUUUUUUCUUUUUUUUUUCC(U24)CUUUUCUUUUU
D85516.1b	5′GGCCAUUC	U16	CUUUCUUCUUU
D89815.1b	5′GGCCAUCCCCUUC	U22	CCUUUUCUUCUUU
EU857431.1b	5′GGCCAUCCUGUUUUUUUCCC(U11)C	U29	CUCCUUUUUUUUUCCUCUUUUUUUCCUUUUCUUUCCUUU
FN435993.1b	5′GGCCGUCCUG(U19)CC	U67	CUUCUUUCUUUCUU
GU133617.1b	5′GGCCAUUUCCUG	U53	C(U17)CC(U20)CUUUCCUUCUUUUUUCCUUUCUUUUCCUUCCUUCUUUAAU
AB520610.1a	5′GGCCAUUCCUG	U16	CUUUUGUUUUUUUUG(U17)CCUUUC(U15)CCUUUCUUCUUUAAU
AF009069.1a	5′GGCCAUUUCCUG	U20	CUUUCCUUCUUUUUUCCUUUCUUUUCCUUCCUUCUUUAAU
AF009070.1a	5′GGCCAUUUCCUG	U34	CUUUUCCUUCUUUUUCCCUUUUUCUUUCUUCCUUCUUUAAU
AF009071.1a	5′GGCCAUUUCCUG	U51	C(U17)CC(U20)CUUUCCUUCUUUUUUCCUUUCUUUUCCUUCCUUCUUUAAU
AF009072.1a	5′GGCCAUUUCCUG(U14)CCC	U37	CUUUCCUUCUUUUUUUUCCUUUCUUUUCCUUCCUUCUUUAAU
AF009074.1a	5′GGCAUCCUG	U64	CUUUUCUUU
AF009075.1a	5′ACACUCCAUUUCUUUUUUUG	U67	CUUUUUCUUUCCUUUCUUUUCUGACUUCUAAUUUUCCUUCUUA
AF009076.1a	5′GUCCUUCUG	U78	CCUUACCCUUUCCUUCUUUUCUUCCUUUUUUUUCCUUACUUU
AF009077.1a	5′GGGUCCCCUUG	U12	CUUUCCUUCUUUCCUUUCCUAAUCUUUCUUUCUU
AF011751.1a	5′AGCCAUUUCCUG	U28	CUUUUUUUUUUUCUUUCCUUUCCUUCUUUUUUUCCUUUCUUUUUCCCUUCUUUAAU
AF271632.1a	5′GGCCAUUUCCUG(U15)G	U55	CCUUUCCUUUUUUUUUUUUUUUCCCUUUUUAU
AJ278830.1a	5′GGCCAUCCUG(U22)C	U17	CUUUUUUUUUCUUCUUUUUCUUUCC(U24)CUUCUUUC
EF621489.1a	5′GGCCAUUUCCUG	U46	CUUUUUCCCUCUUUUUCUUCUCUUUUUCCUUCUUUAAU
AB047639.2a	5′GCUAACUGUUCC	U43	CUUUUUUUUUUUUUUCCCUCUUUCUUCCCUUCUCAUCUUAUUCUACUUUCUUUCUU
AB047640.2a	5′GCUAACUGUUCC	U38	C(U15)CCCUCUUUCUUCCCUUCUCAUCUUAUUCUACUUUCUUUCUU
AB047641.2a	5′GCUAACUGUUCC(U11)C	U27	CCUUCUUUCUUUCUUUCUUACCUUACUUUACUUUCUUUUCU
AB047642.2a	5′GCUAACAGUUUCUC(U13)- CC(U6)AUUUUUA	U25	AUUUUCUUUUCCUUUCUUUCUCACCUUACAUUACUUUCUUUCUU
AB047643.2a	5′GCUAAUUUCCUUAUUG	U19	CUUUCCAUUUCCUUCCUUCUUACUUCACUUUACCUUCUUUCU
AB047644.2a	5′GCUAACUG	U77	CCUUUCCUUUCUUUCUUACCUUACUUUACAUUCUUUUCU
AB047645.2a	5′GCUAACUGUUCC	U70	CUUUCCUUCCUUUCUCACCUUCUUUUACUUCUUUCCU
AF169002.2a	5′GCUAACUG	U45	CUUUUCUUUCCUUUCCUUCUUACUCUACUUUACUUUUUCU
AF169003.2a	5′GCUAACUGUUC	U78	CUUUUCCUUCUUCUUUCUUACCUUAUUUUCCUUCUUUCUU
AF169004.2a	5′GCUAACUG	U30	CUUUUUUUUUCUUUUCUUUCCUUCUUACCUUACUUUACUUUCUUUUCU
AF169005.2a	5′GCUAACUG	U81	CCUUUUUCCUUUUCCUUCUCUUUUUACCUUACUUUACUUUUCUU
AF177036.2a	5′GCUAACUGUCCC	U84	CUUUUUUUCUCUUUUCCUUCUUUCUUACCUUAUUUUACUUUCUUUCCU
AY746460.2a	5′GCUAACUGUCCCUUUUUUUUUG	U30	C(U18)GUUUCUUUUCCUUCUCAUUUCCUUCUUAUCUUAAUUACUUCCUUUCCU
D67095.2a	5′GCUAACUG	U39	CCUUCUUCCUUUCCUUCUUACCUUACUUUAUUUUCUUUCCU
D67096.2a	5′GCUAACUG	U54	CUUUCUUUUCUUUUCUCACCUUACUUUACUUCCUUUCUU
AB030907.2b	5′GCUAGUUUUC	U24	G(U14)CCUCUUUUUCCGUAUUUUUUUUUUUUCCUCUUUUCUU

a DNA sequences were obtained from GenBank, converted to RNA sequences, and aligned to examine sequence variability. Duplicate sequences were removed from the alignment, and sequences are listed as [GenBank Accession #.Genotype].

b Within the 5′arm of the pU/UC tract, genotype 1 nucleotide composition ranged from 12.1–44.4% purine nucleotides; genotype 2 nucleotide composition ranged from 16.7–50% purine nucleotides.

c Within the 3′arm of the pU/UC tract, genotype 1 nucleotide composition ranged from 0 (16 sequences)–11.6% purine nucleotides; genotype 2 nucleotide composition ranged from 5.0–11.4% purine nucleotides.

## Discussion

We have demonstrated that the 34 nucleotide poly-uridine core of the HCV poly-U/UC tract is required for non-self recognition by RIG-I. Interspersed ribocytosine nucleotides between the poly-U sequences in the RNA were also important for optimal RIG-I signaling to the IFN-β promoter. Our RIG-I/RNA binding studies found that RIG-I formed weaker interactions with HCV RNAs lacking poly-U sequences, while the 34 nt poly-U core of the poly-U/UC tract was required to stimulate stronger RIG-I/RNA binding interactions. Additionally, limited-trypsin proteolysis studies revealed that while the RIG-I RD interacts with the 5′-ppp terminus of HCV RNA, interactions between the helicase domain and poly-uridine HCV RNA sequences are required to activate RIG-I signaling. Finally, we found that poly-U/UC RNA variants with high RIG-I signaling activity induced significant anti-HCV responses in cultured cells and also induced hepatic innate immune responses *in vivo*. Together, our studies identify long poly-uridine sequences (>U17) with interspersed ribocytosines as an HCV PAMP motif that drives optimal RIG-I signaling.

Our previous studies also demonstrated the importance of the 5′-ppp for non-self recognition by RIG-I, wherein 5′-ppp was necessary but not sufficient for PAMP activity conferred by HCV RNA [26]. Our current results reveal the additional requirement for the U-core as a non-self signature to demonstrate the combinatorial presentation of multiple non-self motifs within a PAMP RNA. These include 5′-ppp, poly-uridine sequences and arrangements such as interspersed ribocytosine nts, as well as length and certain secondary structures [12], [25], to define an RNA as non-self. Such a requirement for combinatorial non-self recognition by RIG-I serves to provide several checkpoints for immune signaling that prevent spurious recognition of self, thus avoiding autoimmune reactions by requiring PAMP motifs that confer stable RIG-I interactions to induce activation of RIG-I signaling.

RIG-I is maintained in an auto-repressed conformation in uninfected cells [9]. The initial step in RNA recognition and binding likely involves RD interaction with 5′-ppp RNA due to the high affinity of the RD for 5′-ppp moieties [19], which brings RNA structures in close contact with the helicase domains to allow for more specific RIG-I/RNA interactions. Following ligand RNA binding, RIG-I uses ATP hydrolysis to translocate along an RNA [40] wherein upon engaging a PAMP motif it undergoes conformational rearrangements that release the N-terminal CARDs for downstream ubiquitination [41], translocation to mitochondrial-associated membranes [42], and interaction with MAVS to drive IFN expression [3], [4], [42]. Recent RIG-I structural studies have found that a V-shaped linker/pincer/bridging domain connects the helicase domain 2 and the RD, and likely controls RIG-I conformational changes following RNA binding through strong interactions with helix α17 from helicase domain 1 [15], [16], [17], [43]. We found that RIG-I/RNA binding association differences correlated with PAMP activity, revealing that the 34 nt poly-U core of the poly-U/UC tract was required to stimulate potent RIG-I/RNA binding interactions, form contacts with the helicase domain, and activate RIG-I signaling to the IFN-β-promoter.

Our results imply a model of RIG-I interaction with the HCV PAMP RNA in which the RIG-I RD interacts with the 5′-ppp terminus of HCV ligand RNA. While kissing loop interactions between structures in the 5′ and 3′ NTR of the HCV genomic strand RNA [36], [44] would be expected to bring the 5′-ppp into proximity to the poly-U/UC tract for non-self recognition, these motifs are each present in the anti-genomic strand replication intermediate HCV RNA (5′-ppp with poly-A/AG) where they confer PAMP activity through RIG-I recognition [26]. This process of RIG-I binding brings RNA sequence domains into close proximity for PAMP recognition and binding with the helicase domain, thus providing an opportunity for RIG-I to form more stable/specific RNA contacts. ATP hydrolysis allows RIG-I to translocate on the RNA and “scan” for HCV PAMP sequences. Following the recognition of an HCV PAMP motif, defined here as poly-uridine sequences (>U17) with interspersed ribocytosines, RIG-I likely undergoes a final conformational change to activate signaling via the CARDs and drive the innate immune response to infection.

All HCV genomes contain a poly-U sequence in the 3′ NTR; therefore, certain restrictions must exist which prevent viral evolution of this genomic region to mitigate RIG-I recognition. Indeed, previous studies have reported that the poly-U/UC tract is essential for HCV RNA replication [34], [35], [36]. A minimum pU/UC core length of 26 consecutive uridine nucleotides (U26) is required for HCV replication [34], while a core length of 33 uridine nucleotides is necessary for optimal HCV RNA amplification in cell culture [36]. A detailed study by You and Rice (2008) revealed that in addition to the long-range kissing-loop interactions between the NS5B SL3.2 and the 3′SL2 loops in the genomic RNA [36], [44], a poly-U/UC tract with a minimum U-core length of 33 uridines (U33) is also necessary for HCV RNA replication [36]. In addition, viral mutants with truncated poly-U core lengths had impaired replication kinetics (U27 core) or absent replication (U7 or U16 core) until selection for longer U-core lengths resulted in greater replication fitness [36]. The strong selective pressure for a long uninterrupted poly-U nucleotide sequence within the pU/UC tract suggests that this region of the HCV RNA may mediate essential interactions with replication factors, thus explaining the evolution restriction of this viral genomic region. Due to the high fitness cost, HCV is unable to prevent RIG-I recognition via genomic sequence evolution, which makes the HCV poly-U/UC tract an optimal target for non-self recognition. We found that deletion of the U-core resulted in the loss of PAMP activity to drive RIG-I signaling to the IFN-β-promoter, while a U17 core restored some signaling, indicating that RIG-I recognition of the poly-U core can occur at a U-core length below what is required for efficient HCV RNA replication. This restriction also explains why the viral NS3/4A protease has evolved to target the downstream signaling protein MAVS for cleavage in order to suppress the RIG-I pathway and evade restriction otherwise imposed by the innate immune response in HCV patients [45].

HCV encompasses 6 major genotypes and multiple subtypes, and this increased viral diversity results in highly variable treatment outcomes [46]. Standard treatment is currently limited to interferon (IFN)-based therapies, and although two viral protease inhibitors were recently approved for use in humans, these drugs are to be applied in combination with IFN therapy. Overall, treatment with IFN-based therapy results in viral clearance in only 50% of infected subjects, and HCV genotypes 1a and 1b are the least responsive to standard therapies [46], [47], [48]. RIG-I recognition of HCV RNA and subsequent activation of antiviral immune responses may influence the response to therapy, especially in subjects taking viral protease inhibitors where the RIG-I pathway blockade by the viral NS3/4A protease would be temporarily lifted [45]. We examined genetic variability within the poly-U/UC tract of the HCV genome between different viral genotypes using sequences available from the GenBank database. In general, HCV pU/UC genotype 1 sequences contained fewer purine nucleotides than genotype 2 sequences. In addition, nine pU/UC sequences contained a U-core with fewer than 20 consecutive uridine nucleotides, likely representing poorly replicating genomes with decreased fitness. Further studies will help determine whether innate immune activation differs substantially between HCV genotypes and within patient quasispecies populations to impact the outcome of HCV infection and immunity.

HCV evades the host immune response using multiple mechanisms, while host PRRs target critical regions of viral RNA or protein to suppress HCV replication [49], [50]. Understanding the virus-host interface regulating innate immunity against HCV is necessary to develop new therapies and restrict infection. We found that a 5′-ppp and the 34 nt poly-U core within the HCV poly-U/UC tract are required for non-self recognition of HCV RNA by RIG-I. In addition, RIG-I recognition of the U-core within the poly-U/UC tract can activate innate anti-HCV immune responses *in vitro* and hepatic innate immune responses *in vivo*, thus providing a potential target for restricting HCV infection. Similar poly-uridine molecules could be used to induce antiviral immune responses in conjunction with IFN-based and viral protease-targeted therapies to improve HCV clearance in chronically-infected subjects. In addition, vaccine strategies that activate RIG-I signaling pathways may be required to induce appropriate immune responses to RNA viruses, including HCV, which are highly sensitive to IFN and other innate antiviral ISGs in the absence of viral antagonism. Given that RIG-I can activate innate antiviral immune responses upon recognition of poly-uridine sequence motifs, incorporation of poly-U sequences into vaccine vectors could act as an adjuvant to mimic the natural early immune response following virus infection.

## Materials and Methods

### Ethics statement

C57BL/6 mice were housed under pathogen-free conditions in the animal facility at the University of Washington. Experiments and procedures were performed with approval from the University of Washington Institutional Animal Care and Use Committee (IACUC; protocol number 4158-01). Methods for mice use and care were performed in accordance with the guidelines of the University of Washington Institutional Animal Care and Use Committee, and also follow the recommendations in the Guide for the Care and Use of Laboratory Animals of the National Institutes of Health.

### Cells and viruses

Huh7 cells and Huh7.5 cells were cultured in Dulbecco's modified Eagle medium (DMEM) supplemented with 10% fetal bovine serum (FBS) and 100 µg/ml of penicillin and streptomycin. Huh7.5 cells encode a mutant RIG-I protein that cannot signal [33], [39]. The hepatitis C virus (HCV) used in these studies was a cell culture adapted virus that was produced from the pJFH-1 HCV 2a infectious clone as previously described [51].

### Plasmids and proteins

The plasmids pIFN-β-luc, pCMV-*Renilla*-luc [52], and pJFH-1 [51], [53] have been described. The pX-region-c4 plasmid was generated by inserting an HCV Con1 X-region T7 promoter-linked PCR product into the pCR2.1 vector (Invitrogen) as per the manufacturer's instructions. Purified recombinant full-length RIG-I protein was kindly provided by Dr. J. Marcotrigiano (Rutgers University) and was produced as previously described [15].

### RNA methods

All in vitro transcribed RNAs contain a 5′-triphosphate (5′-ppp) and three guanine nucleotides at the 5′ end to enhance T7 polymerase transcription. HCV X-region 5′-ppp RNA was synthesized from a T7 promoter-linked PCR product generated from the pX-region-c4 plasmid using the primers X-regionF (5′-TAATACGACTCACTATAGGTGGCTCCATCTTAGCCCTA-3′) and X-regionR (5′-ACTTGATCTGCAGAGAGGCCAGTATCA-3′). The amplified PCR product was purified by agarose gel extraction using the QIAquick gel extraction kit (Qiagen) as per the manufacturer's protocol. Full-length HCV RNA was produced from the pJFH-1 plasmid (genotype 2a) as previously described [51]. All other 5′-ppp RNA products were generated using synthetic DNA oligonucleotide templates (Integrated DNA Technologies) and the T7 RNA polymerase as described by Milligan *et al.* [38] using the T7 MEGAshortscript kit (Ambion) as per the manufacturer's instructions. Following in vitro transcription, DNA templates were removed with DNAse treatment and unincorporated nucleotides were removed from the reaction using illustra MicroSpin G-25 columns (gel filtration column chromatography, GE Healthcare). RNA was then precipitated using ethanol and ammonium acetate as described by the manufacturer, then resuspended in nuclease-free water. RNA concentration was determined by absorbance using a Nanodrop spectrophotometer. RNA quality (Figure S1) was assessed on denaturing 8 M urea polyacrylamide gels for short RNA transcripts (50–150 nts). Full-length HCV RNA quality was assessed on a denaturing formaldehyde-agarose gel (Figure S1B).

### Luciferase reporter assay

Huh7 or Huh7.5 cells were plated on 10 cm dishes, and 24 hours later cells were transfected with 5.76 µg pIFN-β-luc (firefly luciferase) and 0.24 µg pCMV-*Renilla*-luc (*Renilla* luciferase) plasmids using the FuGENE 6 transfection reagent and protocol (Roche). Transfected Huh7 or Huh7.5 cells were incubated at 37°C for 18 hours, then split into 48-well plates and incubated for an additional 12 hours prior to RNA transfection. RNA transfection was conducted in a 48-well plate format using the *Trans*IT-mRNA Transfection kit (Mirus) as per the manufacturer's instructions. RNA transfection was conducted using either equal numbers of moles of each RNA or 350 ng RNA, depending on the experiment. Following RNA transfection, cells were incubated an additional 18 hours and luciferase activity was measured using the Dual-Luciferase reporter assay system (Promega). All conditions and experiments were conducted in triplicate.

### EMSA

Various amounts of purified recombinant full-length RIG-I protein (0–30 pmol) were mixed with 10 pmol RNA and 10 µl ATPase reaction buffer (20 mM Tris-HCl, pH 8.0; 1.5 mM MgCl_2_; 1.5 mM DTT). Reactions were incubated at 37°C for 15 minutes, then 4× native sample buffer (25 mM Tris-HCl, pH 6.8; 0.02% bromophenol blue; 60% glycerol) was added to samples. Products were separated on a 2% agarose gel (TAE, pH 7.2) and RNA was visualized using SYBR Gold nucleic acid stain (Invitrogen). Gel-shift images were analyzed using ImageJ software (National Institutes of Health), and RNA-protein binding curves were graphed using Prism 5 software (GraphPad).

### Limited trypsin proteolysis of RIG-I/RNA complexes

Various amounts of RNA (0–10 pmol) were mixed with 30 pmol purified RIG-I protein, 2 µl of 5× reaction buffer (20 mM Tris-HCl, pH 8.0; 1.5 mM MgCl_2_; 1.5 mM DTT; 70 mM KCl), 0.67 µl AMP-PNP (10 mg/ml), and nuclease-free water up to 10 µl total volume. Reactions were incubated for 5 minutes at room temperature. Sequencing grade TPCK-treated modified trypsin (Promega) was added to the RIG-I/RNA mixtures at a protease∶protein ratio of 1∶20 (w/w), and the reactions were incubated at 37°C for 15 minutes. Proteolysis was stopped by adding 0.5 µl TLCK (1 mg/ml) and incubating reactions for 5 minutes at room temperature. SDS-PAGE Laemmli sample buffer (Bio-Rad) was then added to the samples and reaction products were analyzed by SDS-PAGE followed by silver stain using the SilverQuest silver staining kit (Invitrogen) as per the manufacturer's instructions.

### ATPase assay

Various amounts of RNA (0–1 pmol) were mixed with 5 pmol purified RIG-I protein in a total of 25 µl ATPase reaction buffer (20 mM Tris-HCl, pH 8.0; 1.5 mM MgCl_2_; 1.5 mM DTT). Reactions were incubated at 37°C for 15 minutes, ATP (Sigma) was added to the reaction mixture at a final concentration of 1 mM, and the reactions were incubated at 37°C for 15 minutes. Free-phosphate concentration was determined using BIOMOL Green reagent (Enzo Life Sciences) in a microplate format and absorbance was measured at OD_630 nm_.

### Immunoblotting and antibodies

Protein extracts were prepared and analyzed by immunoblotting as previously described [52] using antibodies specific to phospho-IRF-3 Ser396 (Cell Signaling Technology), IRF-3 (from A. Rustagi at University of Washington, Seattle), RIG-I (Enzo Life Sciences), ISG56 (from G. Sen at Cleveland Clinic Foundation, Cleveland), and tubulin (Sigma). All secondary antibodies were obtained from Jackson ImmunoResearch, and immunoreactive bands were detected with the Amersham ECL Plus Western Blotting Detection Reagents (GE Healthcare).

### HCV infections

Huh7 cells were plated on 48-well plates and incubated for 12–24 hours at 37°C. Cells were transfected with 350 ng RNA using the *Trans*IT-mRNA Transfection kit (Mirus) as per the manufacturer's instructions and incubated at 37°C for 12 hours. The transfection media was removed and the cells were washed gently with complete DMEM. Transfected cells were then infected with cell culture adapted JFH-1 HCV (MOI = 0.1) in 100 µl total media volume and incubated at 37°C for 3 hours. The virus inoculum was then removed and 300 µl complete DMEM was added, and the cells were incubated at 37°C for 48 hours. HCV-infected cell supernatants were collected and titered on Huh7.5 cells. For the HCV titer assay, Huh7.5 cells were plated on 48-well plates and incubated for 12–24 hours at 37°C, media was removed, and 100 µl of infectious supernatants were added to cells using the following dilutions (no dilution, 1∶2, 1∶10, 1∶100, 1∶1000). Cells were incubated with supernatants at 37°C for 3 hours, the virus inoculum was removed and complete DMEM (300 µl) was added, and cells were incubated at 37°C for 48 hours. Media was then removed and the cells were washed 2 times with phosphate buffered saline (PBS). Huh7.5 cells were fixed with 4% paraformaldehyde for 30 minutes at room temperature. Cell monolayers were permeablized with a solution of PBS/0.2% Triton X-100 for 15 minutes at room temperature, washed with PBS, and then incubated with 10% fetal bovine serum in PBS for 10 minutes. After rinsing with PBS, cells were incubated with a human antiserum specific for HCV (from W. Lee at the Finnish Institute of Occupational Health, Helsinki) for 1 hour, washed three times with PBS, then incubated for 1 hour with donkey anti-human-HRP secondary antibody. Cells were washed 3 times with PBS, then immunoreactive cells were visualized using the Vector VIP substrate kit for peroxidase (Vector Laboratories) following the manufacturer's instructions. Cells were allowed to dry and focus forming units were counted to determine HCV titers in the cell supernatants. All conditions were conducted in triplicate.

### Mice and immunohistochemistry staining

Mouse experiments and procedures were performed with approval from the University of Washington Institutional Animal Care and Use Committee. C57BL/6 mice were transfected via intraperitoneal injection with 200 µg RNA using a lipid-based *in vivo* RNA transfection reagent (Altogen Biosystems), and were euthanized 8 hours later for comparative measurement of mRNA and protein expression. Following systemic PBS perfusion to remove contaminating blood cells, mouse livers were recovered and fixed in 4% formalin solution for 24 hours and immunohistochemistry staining for mouse ISG54 (from G. Sen at Cleveland Clinic Foundation, Cleveland) was performed as described [26] by the Histology and Imaging Core at the University of Washington.

### Real-time PCR

Mouse liver sections were collected following systemic PBS perfusion and soaked in RNAlater reagent (Ambion). Liver sections were homogenized and RNA was extracted using the RNeasy kit (Qiagen). Synthesis of cDNA was conducted using the iScript select cDNA synthesis kit (Bio-Rad) with both oligo(dT) and random primers following the manufacturer's instructions. One-step real-time quantitative PCR was performed with SYBR Green master mix (Applied Biosystems) using an ABI PRISM 7300 Real-Time PCR System. Gene specific primers for mouse *IFN-β*, *CCL5*, *Ifit2*, *ISG15*, and *GAPDH* were purchased from SABiosciences. Results were normalized to the expression of mouse *GAPDH* mRNA.

## Supporting Information

Figure S1 **RNA gel images.** A) In vitro transcribed RNAs were visualized on denaturing 8 M urea polyacrylamide gels. RNA was stained using SYBR Gold nucleic acid stain. B) Full-length HCV JFH1 RNA was visualized on a denaturing formaldehyde agarose gel. RNA was stained using ethidium bromide.
(TIF)
