# Supplementary figures and images for: Uridine Composition of the Poly-U/UC Tract of HCV RNA Defines Non-Self Recognition by RIG-I
Source: PLoS Pathog. 2012 Aug 2;8(8):e1002839. doi: 10.1371/journal.ppat.1002839 (PMC3410852; doi:10.1371/journal.ppat.1002839)

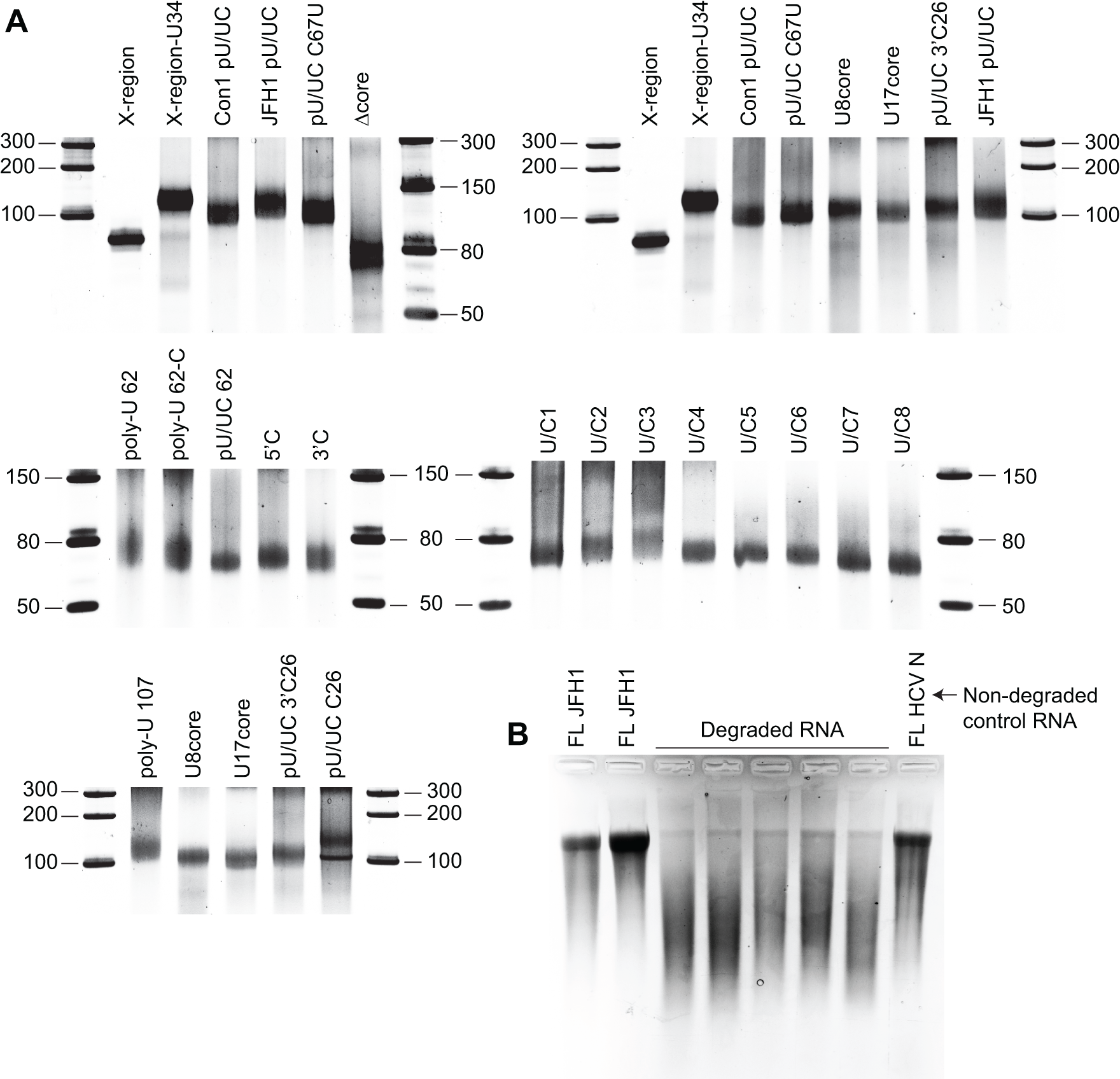

Supplement: Figure S1 — RNA gel images. A) In vitro transcribed RNAs were visualized on denaturing 8 M urea polyacrylamide gels. RNA was stained using SYBR Gold nucleic acid stain. B) Full-length HCV JFH1 RNA was visualized on a denaturing formaldehyde agarose gel. RNA was stained using ethidium bromide. (TIF) [file ppat.1002839.s001.tif]
